# Distinct patterning responses of wing and leg neuromuscular systems to different preaxial polydactylies

**DOI:** 10.3389/fcell.2023.1154205

**Published:** 2023-05-04

**Authors:** Maëva Luxey, Gabriela Stieger, Bianka Berki, Patrick Tschopp

**Affiliations:** DUW Zoology, University of Basel, Basel, Switzerland

**Keywords:** neuromuscular system, limb development, developmental plasticity, polydactyly, Silkie chicken

## Abstract

The tetrapod limb has long served as a paradigm to study vertebrate pattern formation and evolutionary diversification. The distal part of the limb, the so-called autopod, is of particular interest in this regard, given the numerous modifications in both its morphology and behavioral motor output. While the underlying alterations in skeletal form have received considerable attention, much less is known about the accompanying changes in the neuromuscular system. However, modifications in the skeleton need to be properly integrated with both muscle and nerve patterns, to result in a fully functional limb. This task is further complicated by the distinct embryonic origins of the three main tissue types involved—skeleton, muscles and nerves—and, accordingly, how they are patterned and connected with one another during development. To evaluate the degree of regulative crosstalk in this complex limb patterning process, here we analyze the developing limb neuromuscular system of *Silkie* breed chicken. These animals display a preaxial polydactyly, due to a polymorphism in the limb regulatory region of the *Sonic Hedgehog* gene. Using lightsheet microscopy and 3D-reconstructions, we investigate the neuromuscular patterns of extra digits in *Silkie* wings and legs, and compare our results to Retinoic Acid-induced polydactylies. Contrary to previous findings, *Silkie* autopod muscle patterns do not adjust to alterations in the underlying skeletal topology, while nerves show partial responsiveness. We discuss the implications of tissue-specific sensitivities to global limb patterning cues for our understanding of the evolution of novel forms and functions in the distal tetrapod limb.

## 1 Introduction

During vertebrate evolution, adaptations to different styles of locomotion—such as walking, swimming or flying—have resulted in a great diversification of tetrapod limb morphologies. The underlying modifications often occur at the distal-most level, i.e., the autopod, and rely on developmental patterning variations giving rise to different digit numbers and formulas (Zeller et al., 2009; Sears et al., 2018; McQueen and Towers, 2020). For example, modulation of signaling intensities in different molecular pathways—either through temporal changes of signal exposure, overall dose produced or altered sensitivities in the target tissue—have resulted in novel autopod patterns across various clades (Zuniga, 2015). The secreted factor *Sonic Hedgehog* (SHH), in particular, is intricately linked to the developmental specification of digit numbers, as well as evolutionary variations therein (Shapiro et al., 2003; Cooper et al., 2014; Lopez-Rios et al., 2014; Zhu et al., 2022). While the associated changes in the lateral plate mesoderm-derived limb skeleton have been studied extensively, the necessary modifications to match innervation and muscle patterns are only beginning to be uncovered (Tran et al., 2019). Importantly, the three distinct embryonic origins—of skeleton, muscles and nerves—pose a considerable patterning challenge in the developing limb, as these systems mature concomitantly and eventually have to arrive in a stereotypical and functionally interconnected organization (Tsutsumi et al., 2017; Helmbacher and Stricker, 2020).

Limb skeletal muscles derive from myogenic precursors that delaminate from the ventrolateral somitic dermomyotome and migrate into the limb bud in a highly stereotypic and regulated manner (Buckingham et al., 2003). While migrating, these progenitors differentiate into multinucleated muscle fibers that aggregate, and eventually split into individual, anatomically distinct muscles (Christ and Brand-Saberi, 2004; Kardon, 2011; Helmbacher and Stricker, 2020). Concomitantly with the formation of the individual muscle bundles, tendon morphogenesis occurs in close spatial and temporal proximity (Kardon, 1998). Attached to their skeletal elements, contracting muscles then play a decisive role in bone maturation, highlighting the tight functional interactions required for the development of both neuromuscular and skeletal systems (Hosseini and Hogg, 1991; Shwartz et al., 2012; Felsenthal and Zelzer, 2017). SHH signaling affects limb muscle patterning and maturation at multiple levels: for one, muscle mass pre-patterning is closely linked with the development of the muscle connective tissue (MCT), a limb mesenchyme population of lateral plate mesoderm origin (Duprez et al., 1999; Sefton and Kardon, 2019). Moreover, SHH regulates directional muscle cell migration and fiber maturation (Hu et al., 2012) and promotes myogenesis by activating muscle progenitor proliferation (Duprez et al., 1998; 1999; Anderson et al., 2012).

Concurrently with muscle maturation, neurons project their axons to link the central nervous system to the periphery, a prerequisite to execute voluntary motor tasks. In its most basic description, this developing neuromuscular circuit includes a motor neuron cell body in the spinal cord, projecting its axon and connecting to a skeletal muscle fiber in the periphery, with sensory neurons relaying muscle status information back to the central nervous system *via* the dorsal root ganglion (Sharma and Belmonte, 2001; Deries and Thorsteinsdóttir, 2016). Projections from both motor and sensory neurons converge and form a heterotypic nerve trunk at the base of the limb (Honig et al., 1998). From this plexus region, combined sensory-motor nerve projections extend, in an individualized nerve branch manner, to their respective peripheral targets (Hollyday, 1995; Catela et al., 2015). Proprioceptive sensory projections largely follow the motor axons, to connect and deliver muscle feedback to the central nervous system. Nociceptor axons, however, branch off the mixed nerve trunks and form collateral cutaneous projections innervating the skin (Honig, 1982) and their patterning can proceed in the absence of motor neurons (Scott, 1988; Tosney and Hageman, 1989). SHH released from the notochord and floor plate is of central importance for early neuron specification in the spinal cord (Sagner and Briscoe, 2019). However, despite its importance as a guidance cue in other developmental contexts (Zuñiga and Stoeckli, 2017; Douceau et al., 2023), axonal projection patterns in the limb periphery are thought to be only indirectly affected by SHH. Namely, SHH from the limb Zone of Polarizing Activity (ZPA) modulates the composition and distribution of lateral plate mesoderm-derived axon guidance factors in the limb mesenchyme (Bonanomi and Pfaff, 2010; Bonanomi, 2019; Zang et al., 2021).

Here, we evaluate the tissue-specific patterning responses of nerves and muscles to an ectopic source of SHH at the anterior margin of the limb, and test for their ability to adapt to the accompanying changes in digit numbers in two distinct experimental models of polydactyly. We first present a 3D-analysis of the limb neuromuscular system in a genetic model of preaxial polydactyly, the *Silkie* breed chicken with a regulatory allele affecting the limb expression of *SHH* (Dunn et al., 2011; Maas et al., 2011). At both fore- and hindlimb levels, we show that despite the presence of additional cartilaginous structures, the muscle patterns do not follow the appearance of extra digits. However, sensory innervation patterns show plastic responses, albeit to different extents at fore- and hindlimb levels. We then contrast these findings with neuromuscular patterning changes in another type of polydactyly, namely, mirror-image digit duplications following retinoic-acid (RA) bead implantations that also induce an ectopic source of anterior SHH (Pickering et al., 2017; Luxey et al., 2020). Interestingly, compared to polydactyl limbs induced by RA beads, *Silkie* nerve branch organization and muscle patterns appear to be distinct, despite a similar skeletal pattern at hindlimb levels. We discuss how these distinct patterns may relate to differences in SHH signaling strength and/or duration, between the two polydactylies. Overall, our results suggest differential responsiveness of skeletal, muscle and nerve patterning to a global limb patterning cue, with potential implications for the coordinated change and evolution of autopod morphologies and functions.

## 2 Methods

### 2.1 Animals

Fertilized *Silkie* eggs were generously donated by the Zoo Basel, Switzerland. Eggs were incubated, opened and staged as previously described (Hamburger and Hamilton, 1951; Ros et al., 2000). Preaxial polydactyl embryos were dissected after 7–9 days of development. RA-induced mirror-image digit duplication embryos were obtained as described previously (Tickle et al., 1982; Luxey et al., 2020).

### 2.2 Whole-mount immunostaining and tissue clearing

Whole-mount immunostaining on fore- and hindlimbs *Silkie* was performed as previously described (Luxey et al., 2020). Briefly, embryos were fixed in Dent’s fix solution (4:1; methanol:DMSO) for at least 2 weeks and then stored at −20°C until immunostaining. We depigmented the embryos overnight at 4°C in Dent’s bleach solution (4:1:1; methanol:DMSO:H_2_O_2_). After rehydration steps in decreasing methanol concentrations, the samples were blocked and permeabilized before being incubated for a double-immunostaining with primary antibodies against neurofilament (NF200, Sigma, dilution 1:1000) and muscle-specific myosin heavy chain (MF20, DHSB, dilution 1:500) at 4°C with rotation for two nights. After one entire day of washes, the samples were further labeled with secondary antibodies (α-rabbit and α-mouse conjugated; respectively) for two nights at 4°C. Finally, the *Silkie* limbs were cleared using the CUBIC method including a delipidation step in CUBIC 1 solution followed by 2% agarose embedding and incubation in CUBIC 2 solution during 48 h to match refractive indices before lightsheet imaging.

### 2.3 Lightsheet microscopy

Images were acquired on a ZEISS lightsheet 7 microscope using the Zen Blue software 2010 (ZEISS). For acquisition, lasers with fixed wavelengths 488 nm and 561 nm were used. Dual side illumination was applied with the illumination objectives ×5 (ZEISS) and the fluorescence detected with ×5 air detection objectives (PLAN NeoFluar detection optics ×5/0.16 clearing, RI = 1.45). Prior to transfer of the stained and cleared samples into the chamber filled with CUBIC 2 solution, the refractive index (RI) was measured using a Reichert AR7 Series automatic refractometer in order to align manually the lightsheet for optimal acquisition. All images were acquired with sCMOS cameras, pco.EDGE (liquid cooled, 1920 × 1920 pixels, 16-bit readout).

### 2.4 Image processing and 3D imaging

Two image processing methods were used to obtain imaris files (ims) and 3D images. First, TileScan macro (ZEISS) was used to define the tiles of the samples and the step size was optimized by the software. All zeiss.czi lightsheet microscopy files were imported in ArivisVision 4D (Arivis) and stitched together. The files were then exported in .tiff format and finally converted into an Imaris file (.ims). In a second step, the pipeline performing alignment and stitching steps with the Stitchy software was optimized. The output file, in .bigtiff format, was then converted into an Imaris file (.ims) using an Imaris File Converter program. The subsequent analyses were conducted in Imaris 9.0.0 using the “surface” plug-in in order to segment and pseudo-color the nerves and muscles individually.

## 3 Results

### 3.1 Lack of additional muscle bundles in *Silkie* polydactyl limbs

**FIGURE 1:**
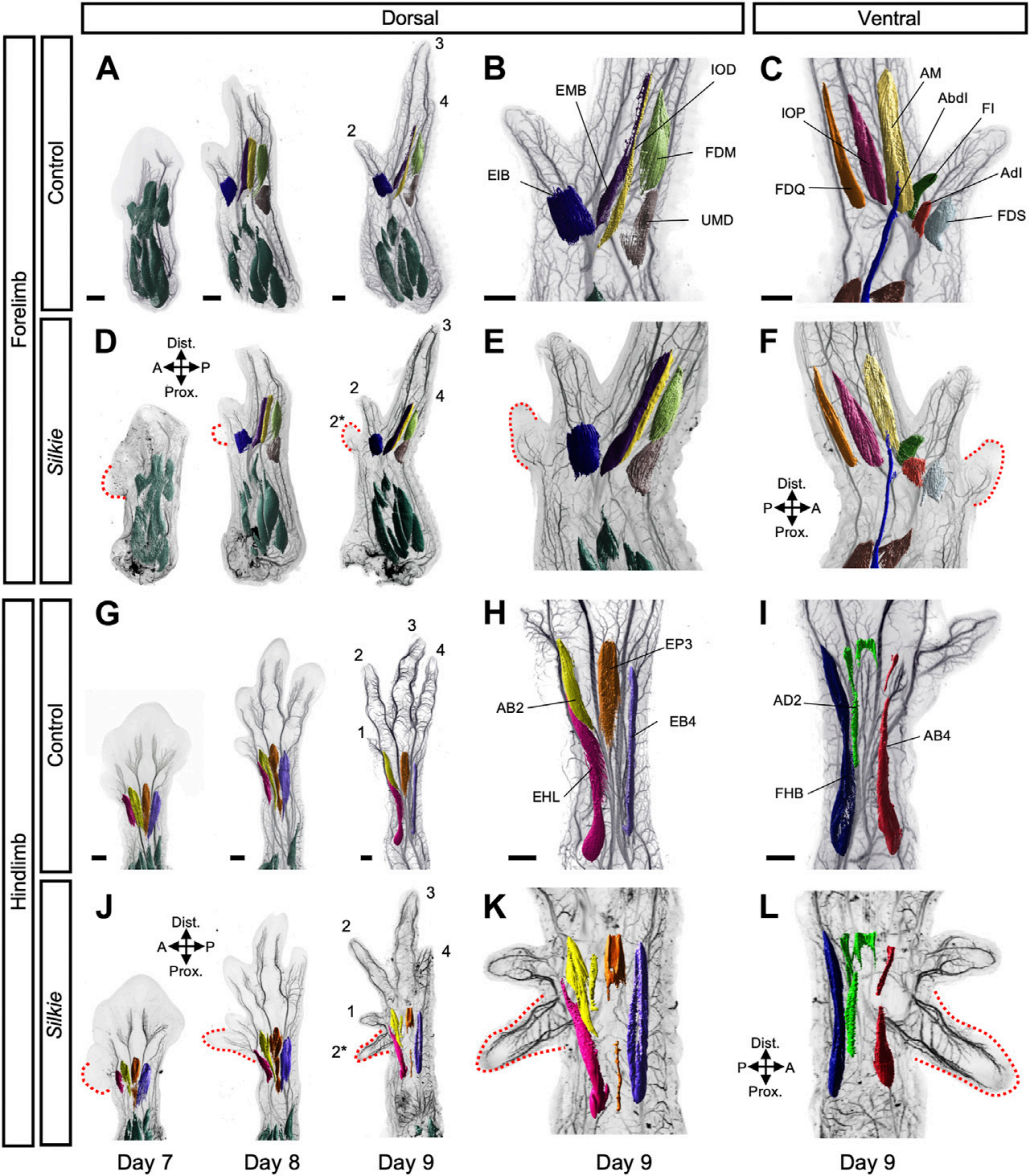
A comparative 3D-analysis of the developing muscles in control and *Silkie* wings and legs.Fore- **(A–F)** and hindlimb **(G–L)** muscle development in control and *Silkie* embryos between day 7 and day 9 of development. Red dotted lines indicate the territories of the forming preaxial extra digits in *Silkie* wings **(D–F)** and legs **(J–L)**. Nerves and muscles were visualized with antibodies against neurofilament (*NF200*) and myosin heavy chains (*MF20*). After 3D-reconstruction, muscle surfaces were rendered using segmentation-based tracing to highlight individual bundles. Individual autopod muscles are pseudo-colored and labeled based on Sullivan, 1962; Kardon, 1998. No individual extra muscles are visible in the preaxial polydactyl territories at day 9 of development, in either dorsal **(E,K)** or ventral **(F,L)** of *Silkie* fore- or hindlimbs (compare to B,H and C,I). Autopod muscle nomenclature: **(B)** Wing dorsal muscles - EIB: *Extensor indicis brevis*; EMB: *Extensor medius brevis*; IOD: *Interosseus dorsalis*; FDM: *Flexor digiti minori*, UMD: *Ulnimetacarpalis dorsalis*. **(C)** Wing ventral muscles—FDS: *Flexor digitorum superficialis*; AdI: *Adductor indicis*; FI: *Flexor indicis*; AbI: *Abductor indicis*; AM: *Abductor medius*; IOP: *Interosseus palmaris*; FDQ: *Flexor digiti quarti*. **(H)** Foot dorsal muscles - EHL: *Extensor hallucis longus*; AB2: *Abductor digit 2*; EP3: *Extensor propius* 3; EB4: *Extensor brevis digit 4*. **(I)** Foot ventral muscles - FHB: *Flexor halluces brevis*; AD2: *Adductor digit 2*; AB4: *Abductor digit 4*. A/P, anterior/posterior, Prox/Dist., proximal/distal. Scale bars represent approx. 500 μm.

To evaluate the potential plasticity of the limb neuromuscular system in response to an ectopic source of SHH and its resulting preaxial extra digit, we performed whole-mount double immunohistochemistry in control and *Silkie* polydactyl wings and legs. With antibodies against neuron-specific intermediate filament protein (neurofilament, NF200) and muscle-specific myosin heavy chain (MHC, MF20), we visualized the developing nerves and muscles, respectively. Using CUBIC clearing, lightsheet microscopy and 3D-reconstructions, we first segmented, pseudo-colored and annotated individual dorsal and ventral muscles bundles (Figure 1; Supplementary Figure S1; for unprocessed muscle images, see Supplementary Figure S2). After migrating into the limb from the dermomyotome, muscle progenitors differentiate and undergo a complex morphogenetic process (Buckingham et al., 2003). At day 7, dorsal and ventral pre-muscle masses in control and *Silkie* forelimbs were still largely fused along the proximal-distal axis (Figures 1A, D; Supplementary Figures S1A, B) (Kardon, 1998; Luxey et al., 2020). In hindlimbs, day 7 pre-muscle masses were already split at the zeugopod/autopod border. At the same time, anterior/posterior cleavages had occurred, resulting in the appearance of individualized muscles at the autopod level first, then at zeugopod levels (Figures 1G, J; Supplementary Figure S1C, D). Thus, muscle splitting in the hindlimb started approximately 1 day earlier than in the forelimb, in agreement with a general developmental heterochrony observed between chicken fore- and hindlimbs (Figures 1A, D, G, J; Supplementary Figures S1A–D) (Bininda-Emonds et al., 2007). From day 8 onwards, each dorsal and ventral muscle bundle acquired its own morphological characteristics, with a uniquely elongated shape and directional positioning (Figure 1; Supplementary Figure S1). Compared to control limbs, all muscles in *Silkie* wings and legs appeared indistinguishable in terms of size, shape and location (Figure 1, compare B,C,H,I to E,F,K,L). Importantly, despite the presence of an extra digit, no additional muscles were observed near the preaxial polydactyl territories (Figures 1D–F, J–L). Indeed, an identical muscular topology manifested itself in *Silkie* limbs, with five dorsal and six ventral muscles in the wing autopod, and four dorsal and three ventral muscles in the foot (Figure 1; Supplementary Figure S1). Given that forming cartilaginous elements are present in the extra digits of both *Silkie* fore- and hindlimbs—at least up to the developmental timepoints we investigated (Arisawa et al., 2006)—this suggested a discrepancy of muscle numbers with respect to the underlying skeletal formula. Collectively, we present a 3D phenotypic description of the developing muscular system in *Silkie* breed polydactyl wings and legs. We uncover a topology that appears identical to control chicken limbs, both in terms of muscle numbers and patterns, despite the presence of additional preaxial digit elements.

### 3.2 Plastic innervation responses to preaxial extra digits in polydactyl *Silkie* wings and legs

**FIGURE 2:**
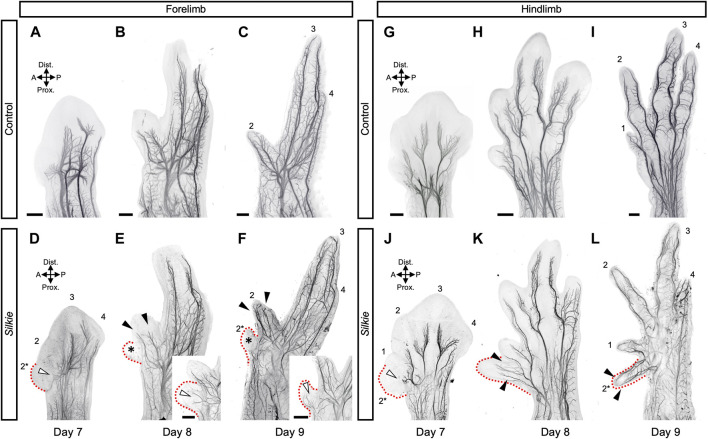
A comparative 3D-analysis of the developing nerves in control and *Silkie* wings and legs.Fore- **(A–F)** and hindlimb **(G–L)** nerve development in control and *Silkie* embryos between day 7 and day 9 of development. Red dotted lines indicate the territories of the forming preaxial extra digits in *Silkie* wings **(D–F)** and legs **(J–L)**. Asterisks highlight the lack of invading nerve branches in the wings preaxial extra digit domain **(E,F)**. White arrowheads highlight the stunted defasciculations at the base of the polydactyl territories **(D,J)** and partial sensory nerve projections occasionally observed in wings (E, F, insets; n = 3/9). From day 8, the preaxial extra digit of the leg shows an innervation pattern similar to the others, with anterior and posterior sensory branches projecting towards the digit tip (K, L; arrowheads). A/P, anterior/posterior; Prox/Dist., proximal/distal. Scale bars represent approx. 500 μm.

We next evaluated potential changes in innervation patterns in *Silkie* wings and legs. At day 7 of development, i.e., approximately 2.5 days after ectopic SHH is induced at the anterior margin of *Silkie* limbs, growing axons reached the distal part of the limb and small branches emerged from the main nerve trunk and started to invade the forming digit territories (Figures 2D, J). However, innervation progress at the base of the polydactyl extra digit territories was delayed, compared to the anterior-most digits in control limbs. Namely, defasciculation and distal extension of sensory nerve branches in *Silkie* fore- and hindlimbs at the anterior margin of the mesopodial-autopodial transition appeared stalled, relative to control limbs (Figures 2D, J, white arrowheads, compare to Figures 2A, G). This was particularly visible at the base of digits 1 and 2* in the *Silkie* hindlimb, where a short, ectopic anterior median fibular nerve bifurcation formed, located at the border of the polydactyl compartment. Its extensions, however, were stunted and not yet starting to elongate towards the distal end as in the control digit 1 (Figures 2G, J; white arrowhead). Later in development, the differences in polydactyl innervation patterns between fore- and hindlimbs became more pronounced. The forelimb in particular showed variability in its sensory nerve projections. While in the majority of *Silkie* embryos digit 2* remained nerveless (Figures 2E, F; asterisks), from day 8 onwards we sometimes observed partial projections—namely, either an anterior sensory branch into the extra digit from the radial motor nerve trunk (n = 2/9) or a posteriorly located cutaneous innervation (n = 1/9) (Figures 2E, F; white arrowheads in insets). The innervation of the neighboring digit 2 appeared unaffected and identical to the digit 2 control at these stages, with both anterior and posterior sensory projections from the radial nerve being present (Figures 2E, F, arrowheads, compare to Figures 2B, C). In the hindlimb, starting from day 8 of development, the polydactyl *Silkie* extra toe was fully innervated, with the typical ‘V-shape’ topology eventually resulting in an anterior and posterior nerve branch (Figures 2H, I, K, L; arrowheads, n = 6/6). Together, our results reveal varying degrees of plasticity in nervous system patterning, between *Silkie* wings and legs, in response to additional preaxial digits. Importantly, the observed nerve patterning changes do not correlate with extra muscle groups, thus uncovering a differential response of nerves and muscles patterns to global limb patterning cues and a preaxial polydactyly in *Silkie* limbs.

### 3.3 Distinct nerve patterns in response to different preaxial polydactylies

**FIGURE 3:**
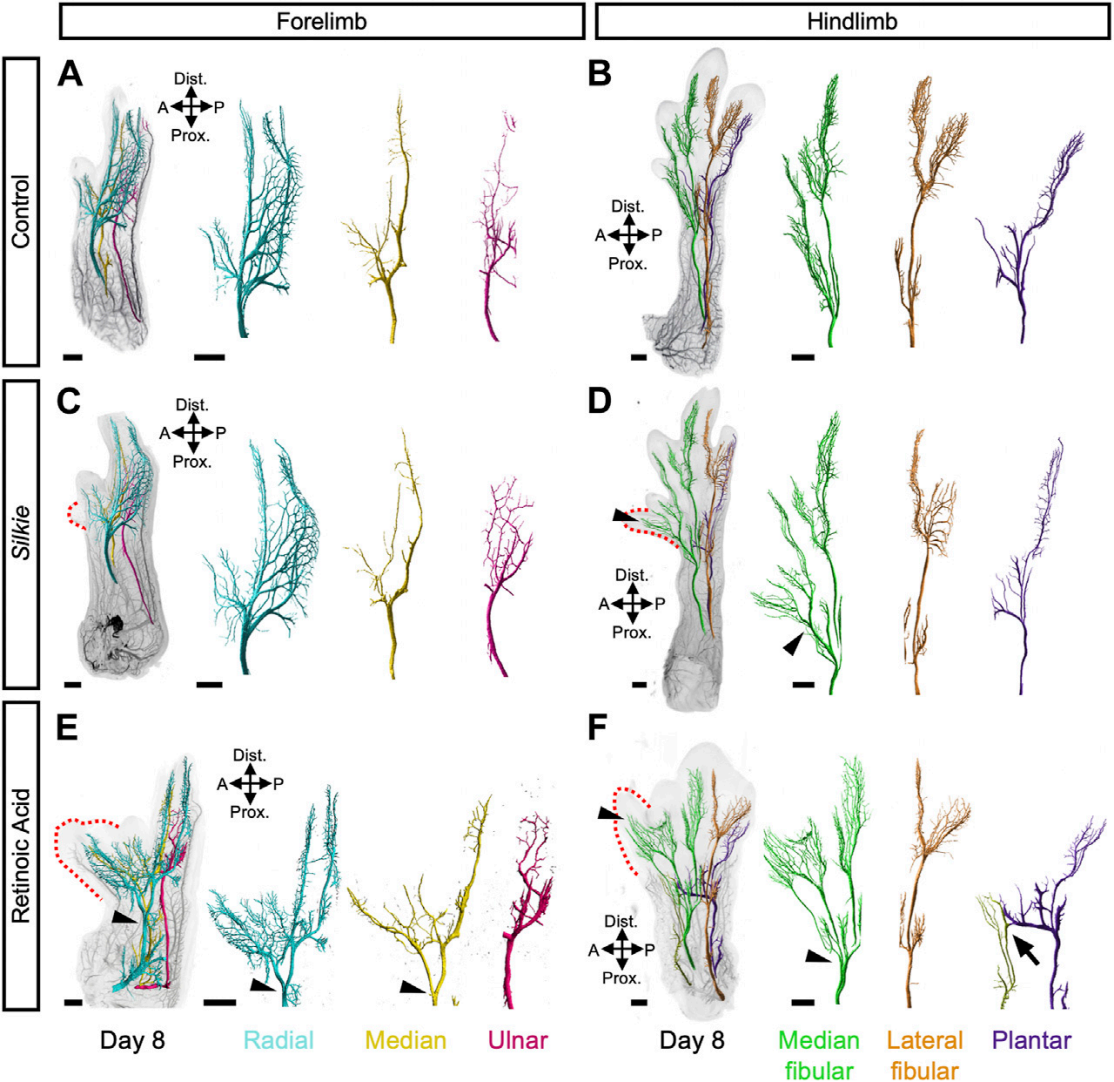
A comparative 3D-analysis of innervation patterns in control, *Silkie* and RA-induced polydactyl fore- and hindlimbs.Dorsal views of 3D-reconstructed developing nervous systems at day 8 of development in control **(A,B)**, *Silkie* **(C,D)** and RA (Retinoic Acid)-treated **(E,F)** limbs. Red dotted lines indicate the territories of the preaxial extra digits **(C–F)**. The three main motor nerves of the wings **(A,C,E)** and legs **(B,D,F)** were surface-rendered using segmentation-based tracing and pseudo-coloring. In the wing, the dorsal radial nerve (cyan) and the two ventral nerves, median and ulnar (yellow and magenta), show a similar branching organization in control and *Silkie* wings **(A,C)**. In the RA-induced mirror-image polydactyl wing, the radial and the median nerves bifurcate (arrowheads) and innervate the extra-digit territory (red dotted line), whereas the ulnar nerve remains unchanged **(E)**. In the leg, the plantar nerve (purple) innervates the ventral part of the leg in an identical manner in control and *Silkie* limbs **(B,D)**, while the fusion with an ectopic projection (light green) is present only in RA-treated limbs (F, arrow). At the dorsal level, the lateral fibular nerve (orange) innervates the last two posterior digits in control, *Silkie* and RA-induced polydactyl limbs **(B,D,F)**. The median fibular nerve (green) shows bifurcation and extra branches innervating the preaxial digit in *Silkie* and RA-induced polydactyl (D, F; black arrow heads), albeit at different proximal-distal levels (D,F, arrowheads). A/P, anterior/posterior, Prox/Dist., proximal/distal. Images are oriented anterior to the right, distal on top. Scale bars represent approx. 500 μm.

Compared to our lightsheet microscopy 3D-analysis of the neuromuscular system in RA-induced polydactylies, various distinct features in the wings and legs became apparent in our *Silkie* data (Luxey et al., 2020). Since no major changes in musculature were discernable in *Silkie* limbs, we focused our attention on the global arrangement of the three major motor neuron-containing nerves, in control, *Silkie* and RA-induced polydactyl limbs at day 8 of development (Figures 3A–F). On the dorsal side of *Silkie* wings, the radial nerve (cyan) followed its stereotypical trajectory from the anterior to the posterior part of the limb (Figures 3A, C). Ventral innervation was provided by the median (yellow) and ulnar (magenta) nerves, both following their usual projection patterns (Figures 3A, C). Hence, an additional anterior digit in *Silkie* wings did not significantly affect the three main motor nerve branches (Figure 3C). This was in stark contrast to RA-treated forelimbs with a mirror-like polydactyly. There, both the radial and median nerves split, approximately midway through the zeugopodal domain, to result in two mirror-symmetrical branches that forked out further to innervate the two autopodial sub-domains in a similar fashion (Figure 3E, arrowheads). Only the ulnar nerve remained unaffected and maintained its control-like branching pattern. In the *Silkie* hindlimb, the lateral fibular (orange, dorsal side) and the ventral plantar nerve (purple) displayed projection trajectories and branching patterns similar to control embryos (Figures 3B, D). The median fibular (green), however, showed an ectopic branching point near the mesopodial-autopodial transition, to provide sensory innervation to the polydactyl digit 2* (Figure 3D, arrowheads). In RA-induced polydactyl hindlimbs, the split in the median fibular occurred at more proximal levels, i.e., within the zeugopod, from where it projected to the single extra digit anteriorly (Figure 3F, arrowheads). Furthermore, on the ventral anterior side of RA-treated hindlimbs an additional, ectopic projection emerged, which fused distally with an otherwise unchanged plantar nerve (Figure 3F, arrow). Collectively, by comparing the innervation patterns in different types of chicken preaxial polydactylies, we show distinct and plastic responses of the projecting main motor nerves. Importantly, even if changes in the underlying skeletal topology are very much alike—as is the case for *Silkie* and RA-treated hindlimbs—this generally does not imply similar responses at the level of innervation patterns, arguing for at least a partial uncoupling of the two systems in their response to limb patterning cues.

## 4 Discussion

Changes in autopod morphologies have paved the way for the execution of highly specialized locomotor behaviors in different tetrapod clades. To do so, the limb skeletal, muscular and nervous systems have to transform in unison, to be functionally interconnected and allow for the controlled movement of the appendage. In this context, digit number variations pose a particular challenge to the underlying embryonic patterning programs, as an entire novel developmental module needs to be accounted for at the level of all three of these systems. Here, we present a lightsheet microscopy 3D-analysis of the embryonic *Silkie* neuromuscular system in fore- and hindlimbs. We show distinct patterning responses of nerves and muscles to a single preaxial extra digit condensation, and contrast them with alterations in mirror-image digit duplications following RA-bead implantation.

The *Silkie* breed is characterized by several distinct morphological features, including a preaxial polydactyly (Dorshorst et al., 2010). This phenotype is associated with a single nucleotide polymorphism in a regulatory region responsible for limb *SHH* expression, a global cue for autopod patterning (Dunn et al., 2011; Maas et al., 2011). As a result, a preaxial extra digit initiates at the anterior margin of both fore- and hindlimbs. Contrary to other polydactylies, we did not find any signs of additional muscle bundles forming, despite the presence of extra skeletal digit elements. In the forelimb, the cartilaginous structures of the extra digit disappear between day 10 and day 11 of development (Arisawa et al., 2006). Nevertheless, that is well after extra muscle bundles have formed in RA-induced polydactylies (Luxey et al., 2020), or when cleavage patterns are completed in general, relative to skeletal maturation (Diogo et al., 2015; Smith-Paredes et al., 2022). Muscle patterns of the *Silkie* autopod are therefore at odds with the underlying skeletal formula, and the ‘nearest neighbor’ rule—i.e., muscles duplicating or splitting to attach to the closest supernumerary skeletal element—appears violated (Diogo et al., 2015; Luxey et al., 2020). However, in addition to skeletal patterning, SHH is also known to impact limb muscle growth, *via* the activation of muscle progenitor proliferation (Duprez et al., 1998; 1999; Anderson et al., 2012). Accordingly, differences in SHH levels, between *Silkie* and RA-induced polydactylies, could account for the observed discrepancies in muscle numbers. Indeed, *Silkie* and RA-induced polydactylies differ both in their expression level and timing of ectopic SHH activation, with RA-induction resulting in a more pronounced and potentially earlier response (Arisawa et al., 2006; Pickering et al., 2017). However, SHH-independent effects of RA on muscle development further complicate direct comparisons between the two polydactylies (Rodriguez-Guzman et al., 2007; El Haddad et al., 2017). Nevertheless, the fact that digit numbers—but not muscles—are identical in both *Silkie* and RA-induced polydactyl hindlimbs argues for a differential sensitivity of the skeletal and muscular systems to a global limb patterning cue, either at the level or the duration of signaling. The molecular basis for such tissue-specific sensitivities, between skeleton and muscles, remains subject for further investigations, e.g., by querying hedgehog receptor expression levels in the respective tissues and their progenitor populations (Feregrino et al., 2019).

Despite the lack of extra muscles, we did observe ectopic nerve projections towards the extra digits, albeit to different degrees in *Silkie* fore- and hindlimbs and RA-induced polydactylies. It has been shown previously that in absence of muscles, motor nerve projections towards them are not maintained (Phelan and Hollyday, 1990). The ectopic digit innervations observed in *Silkie* limbs are thus likely to be sensory in nature, in agreement with their ‘V-shape’ patterns—yet additional stainings would be required, to formally verify this claim (Luxey et al., 2020). The outgrowth of the lateral femoral cutaneous nerve is regulated by the ectoderm (Martin et al., 1989; Honig et al., 2004). A combination of attractive and repulsive factors, in combination with BMP4, instructs this sensory innervation (Honig et al., 2005). Furthermore, sensory projections and the forming vessels interact with one another, and seem to depend on similar local guidance cues at skin levels (Martin and Lewis, 2002; Andreone et al., 2015). This implies that an altered, duplicated patterning topology of the skin, or the underlying vasculature, could be instructive for the ectopic sensory innervation in preaxial extra digits of the *Silkie* breed. Contrary to that, the presence of extra muscles in RA-induced polydactylies is mirrored by the presence and innervation of extra motor nerve projections (Luxey et al., 2020). Given *Sonic Hedgehog*’s role as an axon guidance cue in the central nervous system (Zuñiga and Stoeckli, 2017; Douceau et al., 2023), future studies should also explore the possibility of such ectopic sources of SHH directly affecting these nerve route choices in the limb mesenchyme. All of these observations so far only consider alterations in innervation patterns in the limb periphery. However, these changes are likely to result in concomitant re-wirings and potential cell type or state re-specifications at higher levels of the central nervous system. It will thus be insightful to investigate how motor neurons connecting to extra muscles change their fate (Berki et al., 2023), and what potential impact this might have at the level of the spinal cord, brainstem and upper motor circuits (Mehring et al., 2019; Ruder and Arber, 2019).

Considering the limb neuromuscular system in this intermediary context—i.e., relaying higher order brain decisions to the limb, to execute voluntary motor tasks—is therefore crucial when evaluating evolutionary changes in its developmental patterning. Ultimately, any potential adaptive value of skeletal pattern alterations will rely on the limb still functioning as a coherent unit. To ensure appropriate connections between skeleton, muscles and nerves, a seemingly simple solution would be to have evolutionary changes in skeletal topology dictate exclusively the required pattern alterations of the neuromuscular system. Such sequence of events would also make sense on a developmental timescale, with the early, lateral plate-contained skeletal blueprint providing patterning cues to the incoming muscles, which in turn would instruct the attachments of motor nerves. Despite extensive remodeling in the morphology of their skeletons, however, the evolutionary ground pattern of incoming limb muscles and their first cleavages seem to be conserved among amniotes (Smith-Paredes et al., 2022). Only late changes in autopod muscle splitting, to attach to varying digit numbers, appear distinct in different tetrapod clades. For instance, while previously lost digits in the *Brachymeles* genus of lizards can re-evolve, the reappearance of skeletal structures is not always paralleled by additional muscle bundles (Wagner et al., 2018). This is reminiscent of the situation reported here in *Silkie* polydactyl limbs. Hence, these observations deviate from the most parsimonious patterning solution—i.e., *sensu* “nearest neighbor”, skeleton patterns first, with subsequently developing systems simply adapting to this previously formed topology—and may thereby impair developmental robustness. However, such separated patterning modules are likely to increase the overall evolvability of limb tissue-specific morphologies and functions (Iwaniuk and Whishaw, 2000; Tsutsumi et al., 2017). For example, by partially uncoupling the development of skeletal, muscular and nervous patterns from one another, highly customized autopod architectures with distinct nerve-to-muscle-to-skeleton connections become possible, up to the independent motor control of individual digits in primates.

## Data Availability

The raw data supporting the conclusion of this article will be made available by the authors, without undue reservation.
