# Supplementary material for: Distinct patterning responses of wing and leg neuromuscular systems to different preaxial polydactylies
Source: Front Cell Dev Biol. 2023 May 4;11:1154205. doi: 10.3389/fcell.2023.1154205 (PMC10192688; doi:10.3389/fcell.2023.1154205)
Supplement: Supplementary file 1 [file Image1.pdf]

Supplementary Material

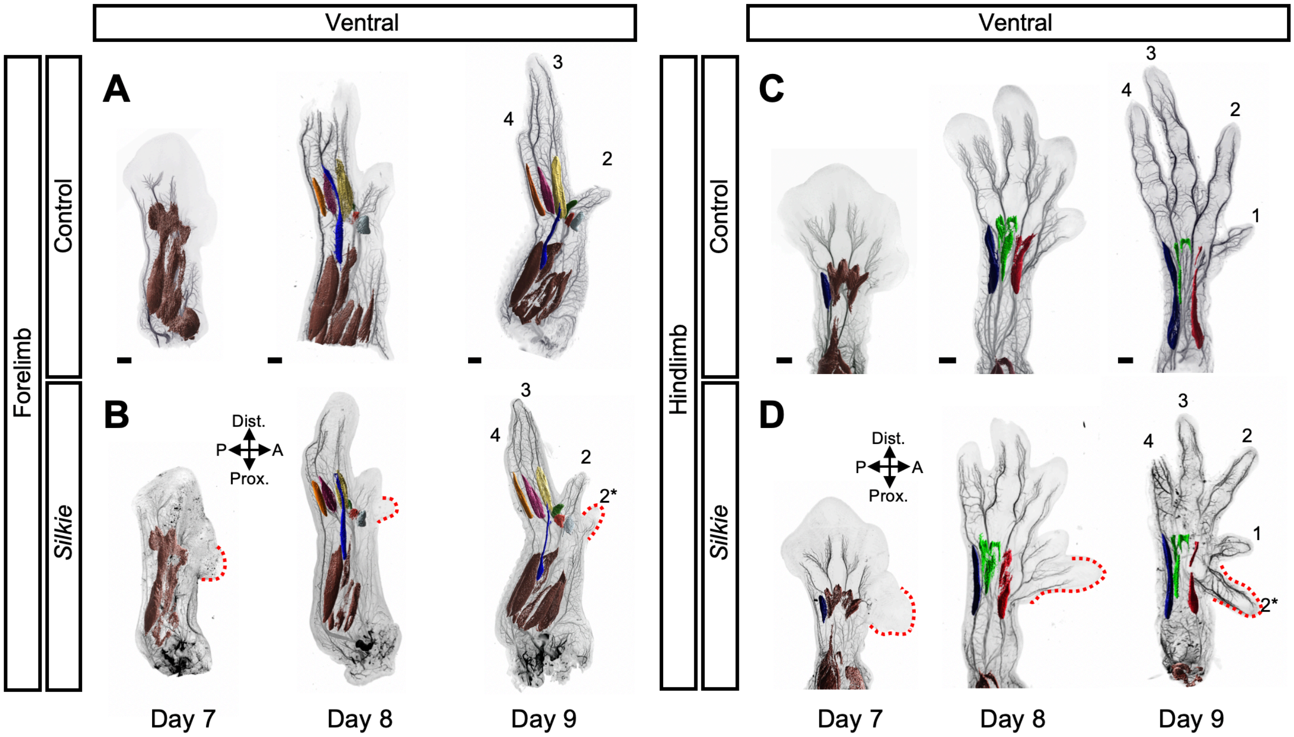

Supplementary Figure 1: A comparative 3D-analysis of the developing ventral muscles in control and *Silkie* wings and legs.
Ventral views of fore- (A,B) and hindlimb (C,D) muscle development in control and *Silkie* embryos between day 7 and day 9 of development. Red dotted lines indicate the territories of the forming preaxial extra digits in *Silkie* wings (B) and legs (D). A/P, anterior/posterior, Prox./Dist., proximal/distal. Images are oriented anterior to the left, distal on top. Scale bars represent approx. 500 µm.

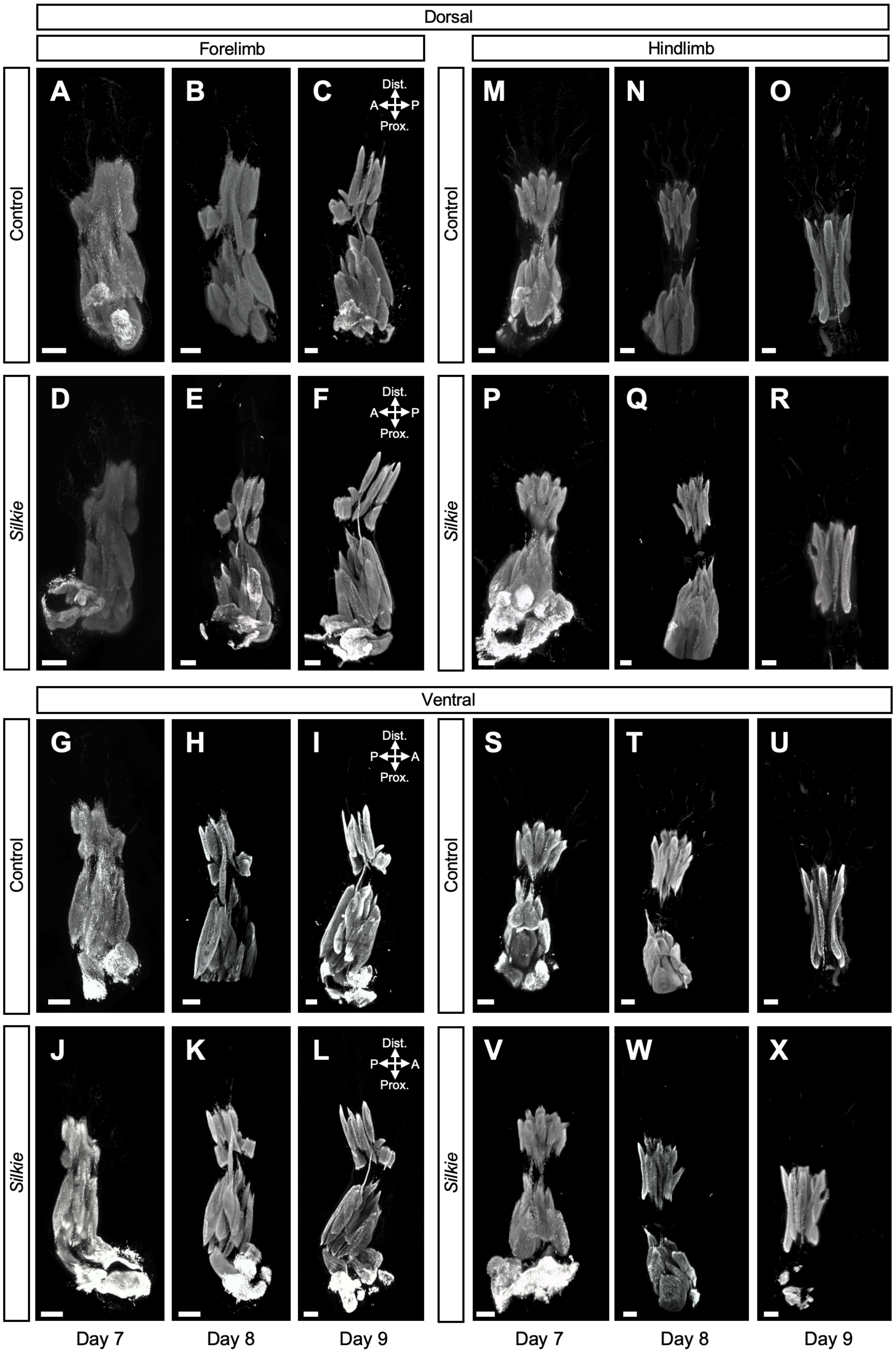

Supplementary Figure 2: Unprocessed images of the developing muscles in control and *Silkie* wings and legs.

Muscle development in control and *Silkie* wings (A-L) and legs (M-X), dorsal (top) and ventral (bottom) views, between day 7 and day 9 of development. Muscles were visualized with antibody against myosin heavy chains (*MF20*). A/P, anterior/posterior, Prox./Dist., proximal/distal. Images are oriented anterior to the right, distal on top. Scale bars represent approx. 500 µm.
